# Storylines: an alternative approach to representing uncertainty in physical aspects of climate change

**DOI:** 10.1007/s10584-018-2317-9

**Published:** 2018-11-10

**Authors:** Theodore G. Shepherd, Emily Boyd, Raphael A. Calel, Sandra C. Chapman, Suraje Dessai, Ioana M. Dima-West, Hayley J. Fowler, Rachel James, Douglas Maraun, Olivia Martius, Catherine A. Senior, Adam H. Sobel, David A. Stainforth, Simon F. B. Tett, Kevin E. Trenberth, Bart J. J. M. van den Hurk, Nicholas W. Watkins, Robert L. Wilby, Dimitri A. Zenghelis

**Affiliations:** 1 Department of Meteorology, University of Reading, Reading, RG6 6BB UK; 2 Lund University Centre for Sustainability Studies, 221 00 Lund, Sweden; 3 McCourt School of Public Policy, Georgetown University, Washington, DC 20057 USA; 4 London School of Economics, London, WC2A 2AE UK; 5 Centre for Fusion, Space and Astrophysics, Department of Physics, University of Warwick, Coventry, CV4 7AL UK; 6 Center for Space Physics, Department of Astronomy, Boston University, Boston, MA 02215 USA; 7 Sustainability Research Institute and ESRC Centre for Climate Change Economics and Policy, School of Earth & Environment, University of Leeds, Leeds, LS2 9JT UK; 8 Willis Re, London, EC3M 7DQ UK; 9 School of Engineering, Newcastle University, Newcastle upon Tyne, NE1 7RU UK; 10 Environmental Change Institute, University of Oxford, Oxford, OX1 3QY UK; 11 Department of Oceanography, University of Cape Town, Rondebosch, 7701 South Africa; 12 Wegener Center for Climate and Global Change, University of Graz, 8010 Graz, Austria; 13 Institute of Geography, Oeschger Centre for Climate Change Research, University of Bern, 3012 Bern, Switzerland; 14 Met Office, Exeter, EX1 3PB UK; 15 Department of Applied Physics and Applied Mathematics and Department of Earth and Environmental Sciences, Columbia University, New York, NY 10027 USA; 16 School of Geosciences, University of Edinburgh, Edinburgh, EH9 3FF UK; 17 National Center for Atmospheric Research, Boulder, CO 80307 USA; 18 Royal Netherlands Meteorological Institute (KNMI), 3730 AE De Bilt, Netherlands; 19 Institute for Environmental Studies, VU University Amsterdam, 1081 HV Amsterdam, Netherlands; 20 Faculty of Science, Technology, Engineering and Mathematics, The Open University, Milton Keynes, UK; 21 Department of Geography, Loughborough University, Loughborough, LE11 3TU UK

## Abstract

As climate change research becomes increasingly applied, the need for actionable information is growing rapidly. A key aspect of this requirement is the representation of uncertainties. The conventional approach to representing uncertainty in physical aspects of climate change is probabilistic, based on ensembles of climate model simulations. In the face of deep uncertainties, the known limitations of this approach are becoming increasingly apparent. An alternative is thus emerging which may be called a ‘storyline’ approach. We define a storyline as a physically self-consistent unfolding of past events, or of plausible future events or pathways. No a priori probability of the storyline is assessed; emphasis is placed instead on understanding the driving factors involved, and the plausibility of those factors. We introduce a typology of four reasons for using storylines to represent uncertainty in physical aspects of climate change: (i) improving risk awareness by framing risk in an event-oriented rather than a probabilistic manner, which corresponds more directly to how people perceive and respond to risk; (ii) strengthening decision-making by allowing one to work backward from a particular vulnerability or decision point, combining climate change information with other relevant factors to address compound risk and develop appropriate stress tests; (iii) providing a physical basis for partitioning uncertainty, thereby allowing the use of more credible regional models in a conditioned manner and (iv) exploring the boundaries of plausibility, thereby guarding against false precision and surprise. Storylines also offer a powerful way of linking physical with human aspects of climate change.

## Introduction

What will the future climate look like? Conventional responses to this question offered by the climate science community (as in the Intergovernmental Panel on Climate Change assessment reports) involve creating large ensembles of simulations of future climate using a variety of global climate models (GCMs). These are used in turn to derive predictions of meteorological fields (e.g., rainfall), expressed in terms of statistical quantities or probabilities of change within the modelled world. The predictions are made under an assumed scenario of future climate forcings (e.g., greenhouse gas emissions) and are called projections. The implications of these projections for particular impacts (such as agriculture) are then investigated by propagating the data through a chain of impact models. Although the forcing scenarios are understood to be non-probabilistic, being subject to human actions, it is standard practice to represent the physical aspects of climate change probabilistically (Swart et al. 2009).

As the scientific and policy discussion shifts from whether anthropogenic climate change is real to the evaluation of potential regional impacts and response options, the limitations of these probabilistic approaches to the physical aspects of climate change are becoming increasingly apparent (Kennel et al. 2016). Climate models have structural errors, many of which are shared, which challenges a probabilistic interpretation of multi-model ensembles (Knutti et al. 2013). In contrast to weather forecasting, the shortness of the observational record from a climate change perspective makes it very difficult to *directly* assess the reliability of future climate projections (Parker 2010). As regional climate phenomena such as storm tracks respond differently to climate change in different models (Shepherd 2014), a multi-model mean can lead to a washed-out response that does not correspond to any model simulation. Effective bias correction of multivariate relationships, such as those involved in compound events, requires vast amounts of data that may not exist. In any case, it is not known how to correct model biases in simulating climate *changes* (as opposed to simulations of the present climate state) (Maraun et al. 2017). Estimates of uncertainties at the regional scale can quickly accumulate to a point where this knowledge hinders rather than supports scenario-led climate adaptation decision-making (Wilby and Dessai 2010).

Alternative approaches are thus emerging that do not seek to quantify probabilities, but instead to develop descriptive ‘storylines’, ‘narratives’ or ‘tales’ of plausible future climates. We broadly refer to these as ‘storyline’ approaches. Whilst there has been variation in the use of these terms, there are some common characteristics: in particular, the emphasis on qualitative understanding rather than quantitative precision, and the acceptance that storylines are not probabilistic. Here, we define a storyline as a physically self-consistent unfolding of past events, or of plausible future events or pathways. (A trend can be considered a long-lasting event.) As no a priori probability of the storyline is assessed, it is not a prediction. Emphasis is placed instead on understanding the driving factors involved and the plausibility of those factors (or of changes in those factors). Typically, more than one storyline is considered, to explore multiple plausible futures. However, we also include past events, because historical events are not simply single data points but involve detailed stories which can be unpacked (March et al. 1991). Woo and Johnson (2018) have argued that the fact that the past is now fixed should not blind us to the usefulness of past sequences of events as available realisations of the time evolution of systems, asserting that scenarios derived this way can be ‘counterfactual histories of the future’. Inspired by the viewpoint of statistical physics they advocate that ‘to fill gaps in knowledge of rare extreme events without waiting an inordinately long time for further occurrence, historical disasters can be used much more extensively as a currently available test laboratory for scenario discovery’.

**Fig. 1:**
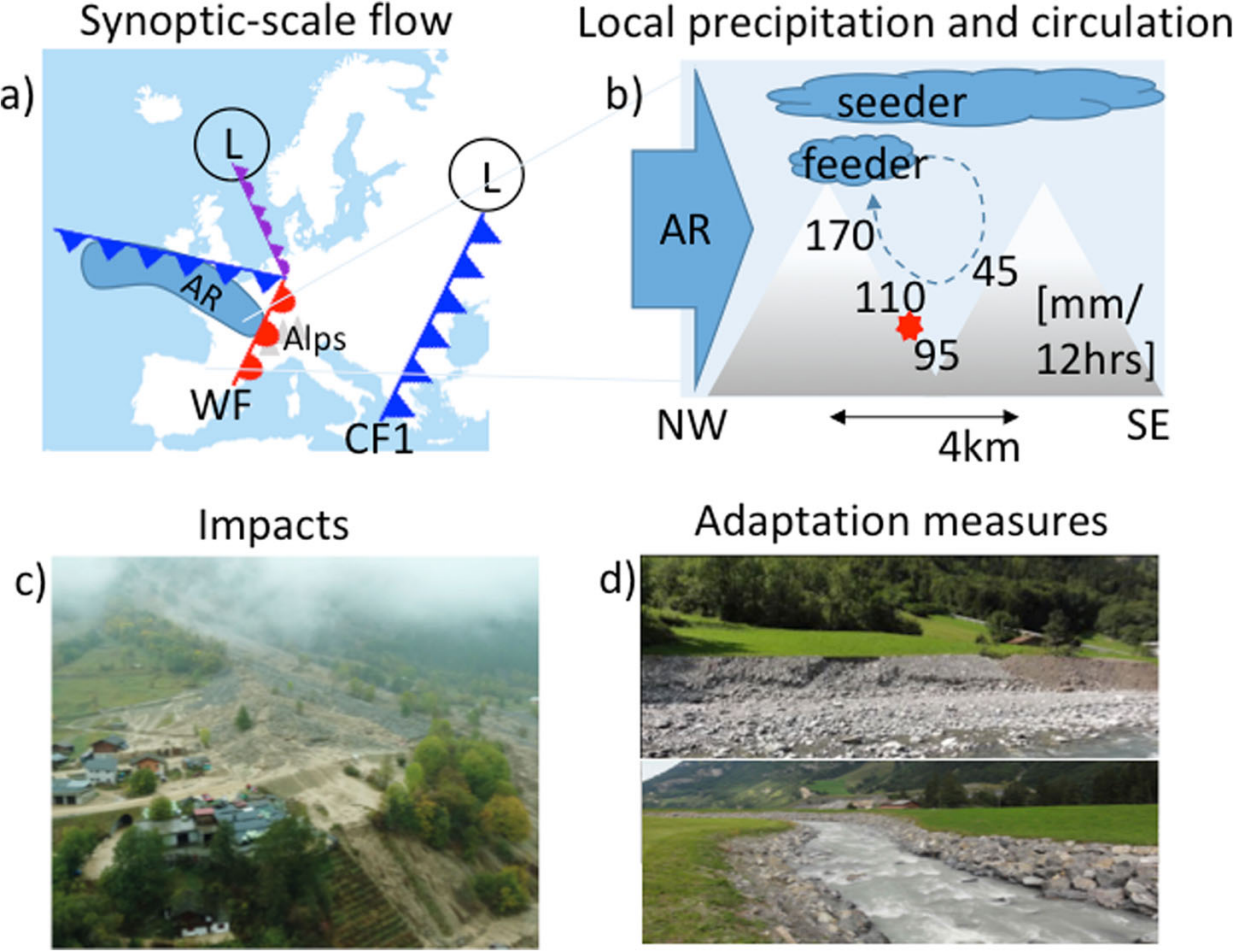
**a** Schematic depiction of the synoptic-scale weather situation over Europe on 10 October 2011 at 00 UTC. A warm front (WF) is located over the Alps; behind the WF, an atmospheric river (AR) reaches the western Alps from the northwest, providing a supply of moisture to the Alps. The cold front (CF1) over Greece had crossed the Alps 2 days earlier, providing heavy snowfall. The L symbols indicate the locations of the low-pressure centres associated with the two fronts. **b** Schematic cross-section (northwest to southeast) across the Lötschen Valley, Switzerland, illustrating the strong contrast in precipitation between the two sides of the valley; the precipitation values [mm/12 h] indicate the accumulation at several locations between 00 UTC and 12 UTC on 10 October 2011. The solid blue arrow indicates the atmospheric river (AR) and the dashed line the cavity circulation, resulting in a feeder cloud. **c** A mud flow blocking and damaging the only road into the Lötschen Valley, located at the red star in panel **b**. Figure from Bern cantonal police, used with permission. **d** Examples of adaptation measures implemented after the flooding: (top) a broader river bed gives the river more space to flood areas where no infrastructure or people can be harmed and (bottom) higher dams. From https://sites.google.com/a/gymneufeld.ch/hochwasserschutz-in-mitholz/schutzbauten-frueher-und-heute/neue

An example of such a storyline is provided by the rain-on-snow event in the Swiss Alps that occurred on 10 October 2011, which led to severe flooding and mudflows (Fig. 1). On 8 October 2011, a cold front reached Switzerland, and in the following 2 days, sustained northerly winds resulted in substantial snow accumulations in the Alps. This was followed by the passage of a warm front on 10 October 2011, combined with an atmospheric river—an elongated and narrow area of very high atmospheric moisture transport—flowing directly into Switzerland. The warming together with the large moisture transport against the Alps resulted in snowmelt together with large amounts of rainfall in the western Swiss Alps (Rössler et al. 2014; Piaget et al. 2015). The combined water from the snowmelt and the rainfall led to flooding and the formation of mudflows in the Kander and Lötschen valleys. The effects were extremely local, as the southeastward-facing slopes received more than three times the precipitation amounts that fell on the northwestward-facing slopes, because of orographic effects (Rössler et al. 2014). The mudflows on the southeastward-facing slopes and the floods had significant impacts on the local transportation infrastructure, blocking roads and a tunnel. A mudflow dyke was subsequently built to protect the tunnel damaged in the event from future similar events, and the river morphology was modified to reduce flood-related risk.

This storyline is of a past event, and the actions taken in response thus fit the most common historical pattern in which mitigating actions against possible future events of a given type are taken only after an event of that type has occurred, as discussed further below. Preparation for future events could, however, be built on a storyline based on this event (or ones of a similar type, real or imagined) combined with climate change information. For example, the increased moisture content in a warmer atmosphere would lead to increased moisture transport against the Alps given the same flow configuration. Climate change can be expected to decrease the probability of such rain-on-snow events in the autumn, but to increase it in the winter. This change in seasonality could matter for impacts since the ground is potentially frozen during winter, which would result in increased direct surface runoff rather than infiltration of water into the soil, and hence more flooding. On the other hand, the snowpack is likely colder during winter and will thus require more energy input to be heated to the melting point, which could reduce the amount of flooding. Ways to develop such future storylines are discussed in the body of the paper.

There are many references to storylines, narratives, and scenarios in climate change literature, and the terms are sometimes used interchangeably. The term ‘narrative’ is often used by social scientists to characterise peoples’ views, understandings or perspectives. Narrative analysis is used to investigate climate change discourses and the framing of climate change by the media, policy-makers or other stakeholders. Whilst these are all important aspects of climate change research (Fløttum and Gjerstad 2017), our focus here is on the rather different task of constructing storylines to represent uncertainty. The term ‘scenario’ is often used in decision-making to represent an imagined future, with reference to the scenario technique of Herman Kahn and colleagues (Kahn and Wiener 1967). The emphasis on self-consistency and plausibility, and the fact it is not a prediction, has much in common with storylines, as does the benefit for risk awareness (Lempert 2013). Indeed, the term storyline is often used in this context, as the part of a scenario where the future is described by words and not numbers (Alcamo 2008). Within climate change science, the term scenario is generally associated with shared socio-economic pathways and their associated climate forcings, where the uncertainty lies in the domain of human choices, and concerns the future. Our focus here is instead on uncertainty in the *physical* aspects of climate change (i.e. for given climate forcings), including consideration of past events.

Storylines can be perceived as anecdotal and thus unscientific. It is, therefore, important to understand their basis and how they contribute to representing and communicating uncertainty in the physical aspects of climate change. We identify some of the main ways in which the storyline concept is being used, structuring our evaluation around a typology of four reasons for taking a storyline approach.

Figure 1 shows how all four reasons are manifest in the consideration of the Swiss rain-on-snow event discussed above; more examples, with additional detail, are provided within each section. The flooding and mudflows (panel c) improve risk awareness (Sect. 2) by providing a salient event, moreover one that was not forecasted. Strengthened decision-making (Sect. 3) is evident in the development of adaptation measures (panel d). The synoptic-scale weather setting (panel a) provides a physical basis for partitioning uncertainty to assess future risk, including the distinction between thermodynamic (warmth and moisture) and dynamic (wind) factors (Sect. 4). Finally, the strong enhancement of the precipitation on the southeastward-facing slopes of the valley was due to a very local cavity circulation and associated ‘seeder-feeder’ effect (panel b), a meteorological phenomenon not captured by the weather forecast models—which is why the event was not forecasted—and which thereby explores the boundaries of plausibility relative to what is currently represented in the models (Sect. 5).

## Improving risk awareness

Two human tendencies are intuitively evident in how we think about risk. It may be useful to classify them using a distinction made first by Tulving (1972, 2002) about memory: knowing facts (semantic) versus reliving events (episodic). Since then, it has become clear that episodic memory has a role in anticipating the future (Schacter et al. 2007), and new neuroscientific discoveries are giving a picture of constructive memory and episodic future simulation, which act as warning bells, and help us to conceive of possible extreme phenomena. Considered within this broader context, the conventional approach to climate change risk is semantic (e.g., what is a 1 in 1000 year event?), whereas storyline approaches are episodic (e.g. have we seen this before; and if so, what might the next event be like?).

Behavioural psychology shows that humans have difficulty responding rationally to risks from events that are outside their experience, even when accurate quantitative information on these risks, and the benefits of rational mitigation actions, is available. Even when given such information, we act as though the probability of a bad outcome is less than it really is if an event of that type has not happened to us recently (or ever), and more probable than it really is if it has. This asymmetrical response is known as the ‘availability bias’ (Kahneman 2011). Essentially, we—even those with quantitative scientific training—are more likely to respond to episodic than to semantic information.

The availability bias is apparent in the history of physical infrastructure measures to mitigate natural disaster risk, which shows that such measures are often taken in direct response to disasters that have just occurred. This was the case with the North Sea flood of 1953, which led the Netherlands to develop the Delta Works and the UK the Thames barrier. A more recent example is Hurricane Sandy in New York City (Sobel 2014). Reports commissioned by state and local government had shown as early as 1995 that the city’s transportation systems were highly vulnerable to storm surge flooding from a potential hurricane, outlining in detail the specific facilities most at risk (U.S. Army Corps of Engineers 1995). Subsequent studies refined the analysis, showing, in particular, the vulnerability of the subway system (Jacob et al. 2001, 2011). These studies had characteristics of a storyline, simulating flooding from specific hypothetical storms—and in the case of the later studies, adding sea level rise in varying amounts according to specific future scenarios. Recommendations made by these studies for investments to storm-proof infrastructure were not followed; to cite one egregious example, a new subway station at South Ferry, the southern tip of Manhattan, was opened in 2009 at a cost of approximately $550M. No substantive flood control measures were incorporated into the station’s design, despite the fact that the station’s location—and especially, its elevation—was essentially the same as that of the old station just adjacent, and that the 1995 report had highlighted that station as being at high risk for flooding. The station did in fact flood during Sandy, and returned to operation only in 2017, 5 years after the storm, at an expense comparable to the initial construction. As in the case of the 1953 North Sea flood, much more substantive investment in flood control has been made since the storm.

These examples are representative of a broader truth that threats from natural events sufficiently rare as to be outside the living memory of local populations and decision-makers do not so readily motivate investments in physical infrastructure (or other costly measures, such as retreat) that would reduce risk, despite good semantic understanding of the case for such investments, because the necessary episodic understanding is not present. In the best cases, scientific information well in advance of an event has led to (less costly) efforts to improve emergency management procedures. This appears most likely to happen when the scientific information is made available in sufficiently episodic form, i.e. as a storyline. This occurred in the case of Sandy in New York City; whilst the studies of the city’s storm risk did not lead to much investment in infrastructure, they did inform the efforts of city, state and federal agencies just before, during and after the disaster. The subway system again provides an illustrative example, here positive rather than negative. Understanding of the likely flooding of the subways led the Metropolitan Transit Authority to remove electrical signal equipment from tunnels likely to be inundated, so that the equipment itself was not damaged and could be reinstalled relatively quickly once the water was pumped out after the storm. Arguably, this action allowed most of the subway system to begin functioning again within a week, whereas it could have taken a month otherwise (New York Times 2013). Something similar arguably occurred in the case of a possible earthquake and tsunami in the Pacific Northwest of the USA, where a popular magazine article (Schulz 2015)—augmented, perhaps, by wide awareness of the 2011 Fukushima event in Japan—led to organised preparation efforts by the federal government (FEMA 2016).

Climate change is essentially similar. The best scientific information predicts a future fundamentally different from the past, but even those who are aware of the science have difficulty prioritising actions precisely because of the unfamiliarity of that future (Weber 2006). Here, scientifically constructed storylines help as a complementary approach to raise risk awareness, by incorporating episodic information and making the predicted future more tangible (e.g. Matthews et al. 2016, 2017).

This is the approach advocated by Hazeleger et al. (2015), who construct storylines (called tales) of future weather that illustrate the implications of climate change for real-life high-impact weather events. This is done by mapping historical or hypothetical events onto future climate conditions. Examples of the approach include rerunning weather episodes in a limited-area weather-prediction model with elevated temperatures as boundary conditions (Attema et al. 2014; Prein et al. 2016), diagnosing unprecedented storms in future climate integrations (Haarsma et al. 2013) and exploring multivariate drivers of local extreme water levels (Van den Hurk et al. 2015). That such ‘simulated experience’ is more effective at conveying risk than statistical characterizations has been recognised more widely in the decision-making literature (Hogarth and Soyer 2015), building on insights from experimental psychology (Gigerenzer and Hoffrage 1995).

There is also the issue of how to communicate the storyline. Well-designed board and card games can offer an immersive experience and tell stories and have been used to aid development decisions (Tint et al. 2015), kick-start discussions on the effects of climate change (Chuang 2017) and inform participants about climate change (Reckien and Eisenack 2013), although most games focus on national-level policy makers (e.g. Matzner and Herrenbrück 2017). The Red Cross Climate Centre (http://climatecentre.org/resources-games/) has developed many simple games to aid stakeholders’ understanding of different humanitarian issues. One way to engage infrastructure owners and managers with potential future climate change could be to produce a deck of cards with each card containing a simple textual description of a weather or climate event. The deck of events is a storyline of a potential future climate; different decks could be constructed to represent different storylines, e.g. with different frequencies of damaging extreme events. The participants then discuss the potential impact of each event on their infrastructure, how the impact of that event on other infrastructure might affect them and how they might mitigate the damage from the event. This allows decision-makers to understand how potentially cascading events might affect their infrastructure, including the cumulative impact of multiple events. The participants would work through the card deck discussing each event, observed and supported by a facilitator. Importantly, the participants, like in reality, would not know what events are still to come. At the end of the deck, the facilitator would then discuss with the participants which events, or combination of events, mattered to them and for which ones damage mitigation would require substantial investment. It is for those events that subsequent risk assessment could be made.

The dialectic between semantic vs episodic knowledge seems related to that between the invisibility vs visibility of climate change (Rudiak-Gould 2013). The orthodox climate science view would be that climate change is invisible to the naked eye, as it is inherently a statistical concept (semantic knowledge). However, many would argue that some aspects of climate change are also visible, as in the case of many of the observed cryospheric changes such as melting of glaciers and loss of summertime Arctic sea ice (episodic knowledge). If an observed change is sufficiently large that even a single occurrence is attributable (Shepherd 2016), and if it is visible to the naked eye (as opposed to something that can only be measured), then visibility of climate change can be reconciled with the statistical perspective. In a similar way, storylines allow episodic knowledge of climate change to be set within the context of semantic knowledge.

## Strengthening decision-making

Few societal decisions are driven solely by, or are framed only by, concerns about climate change, but rather by sustainable development more generally. Storyline approaches acknowledge this context by communicating the potential consequences of climate change in ways that are relevant to the specific decision and the specific decision-maker (Hazeleger et al. 2015). They are also naturally suited to non-probabilistic decision-making frameworks, which seek adaptation options that are robust within a context of deep uncertainty (Kalra et al. 2014; Simpson et al. 2016). Storylines allow a way to explore such uncertainties in a traceable and physically plausible manner (see also the discussion in Sect. 5 concerning sea-level risk in the Netherlands). It is important to undertake such analysis using an iterative process in which decision-maker concerns and interests shape the scientific research and modelling in a process of co-production (Kalra et al. 2014; Bhave et al. 2018).

An example of such an iterative process is provided by an analysis of the physical and legal dimensions of water diversions from the Upper Colorado River Basin under storylines of climate change (Yates et al. 2015). In this case, three regional climate change simulations were used to stress test a model of the water supply system with and without an adaptation option. Stakeholders were engaged from the outset in the development of plausible storylines that captured both the direct and indirect consequences of climate change on the headwater areas. Here, ‘plausible’ meant internally consistent storylines of regional climate selected from within (rather than at, or beyond) the bounds of available climate model ensembles, *and* co-dependent exogenous factors that potentially affect water supply security over the planning horizons. For instance, when the ‘hotter and drier’ storyline was imagined, then water model parameters reflected possible hydrological changes associated with dust on snowpack, beetle infestation, or wildfires destroying forested areas. Stakeholders also specified metrics of water system behaviour and state relevant to their risk management context. In this way, both water managers and researchers arrived at a shared understanding of the system vulnerabilities and the extent to which the specified climate risks could be managed using a legal adaptation instrument.

Storylines are especially effective for considering risks from compound extreme events, namely those where severe impacts are triggered by the interaction of more than one variable. Examples include drought with heat waves (Ciais et al. 2005), windstorms with heavy precipitation (Martius et al. 2016), and fluvial flooding with storm surges (Kew et al. 2013). Figure 1 provides another example. Neither variable itself needs to be very extreme but the combination results in severe impacts (Leonard et al. 2014). To study compound extremes statistically, long time series and refined statistical methods such as copulas are needed to capture and quantify the inter-dependence of the variables. A storyline approach is a very attractive complementary method to address compound extremes as it allows one to define very complex events starting from the impacts in a very flexible way, including spatial and temporal dependence (clustering) of extremes, which can generate the impacts (e.g. Muchan et al. 2015). Storylines based on impacts can then be assigned a categorical plausibility using statements like ‘worldwide at least one similar event occurred before’. The Swiss Federal Office for Civil Protection uses such an approach to prepare for a broad range of perils, including atmospheric hazards, but also technical and social threats (BABS 2013). For each peril, they define three storylines with increasing severity and impacts.

Another emerging application of the storyline approach, although very much in its infancy, is in the insurance sector. The insurance and reinsurance industries are slowly beginning to acknowledge the impacts of climate change on worldwide insured losses (Lloyd’s of London 2014). Having a robust understanding of possible catastrophe losses, particularly of the extreme catastrophes, is essential for any insurance/reinsurance company’s internal capital management, definition of own risk appetite and solvency (EIOPA 2016). The main tools for assessing such risk from natural perils are catastrophe models. By nature, catastrophe models are built to estimate *probabilistic risk*, through the extrapolation of the limited observed record, in order to estimate a range of *potential* catastrophes, their associated probabilities and corresponding losses (Grossi and Kunreuther 2005). However, the same models can be and frequently are used to estimate *deterministic risk* from various storm scenarios. As such, models are run to compute possible losses from a relevant historical event or a modified version of the historical event, for a given portfolio (Woo et al. 2017). This storyline approach of assessing risk for insured properties is an extremely useful method of stress-testing the client’s exposure to various weather and climate conditions.

## Partitioning uncertainty

Many studies have shown that the atmospheric response to climate change can be usefully understood as a combination of what might be called a thermodynamic component (surface warming, moistening, melting of ice) and a dynamic component (changes in atmospheric circulation) (e.g. Deser et al. 2014). The distinction is not precise because both components of the climate system are coupled; in practice, either the dynamic component is defined as the component congruent with internal variability (Deser et al. 2004) with the thermodynamic component obtained as a residual or (especially for precipitation) the thermodynamic component is defined as the component obtained in the absence of changes in circulation, with the dynamic component obtained as a residual (Pfahl et al. 2017).

The reason the distinction is useful is, first, that there is a striking contrast between the degree of confidence we have in the two components (Shepherd 2014; Trenberth et al. 2015). Thermodynamic aspects of climate change are generally robust in theory, observations and models (although there is substantial quantitative uncertainty associated with climate sensitivity). In contrast, dynamic aspects are not anchored in accepted theories, have not yet emerged in observations and diverge between models (Shepherd 2014). Second, model uncertainties in climate sensitivity and in dynamic aspects of climate change (after scaling by global mean warming) appear to be uncorrelated (e.g. Zappa and Shepherd 2017), which makes sense as they are associated with different aspects of model error. The distinction between thermodynamic and dynamic aspects of climate change provides an alternative to the usual approach of considering ensembles of model simulations, which mix together uncertainties in the two aspects, and may help to provide information on where the largest uncertainties lie (e.g. Pfahl et al. 2017). In particular, storylines can be considered for each aspect.

Understanding the role of climate change in weather and climate extremes is of considerable societal relevance. This is not straightforward to resolve, since extreme events always result from an intersection of natural variability of some sort, riding on and augmenting global warming effects, and every extreme event is unique (NAS 2016). The conventional approach to this question focuses on changes in probability but does not deal satisfactorily with the unique nature of each event (Shepherd 2016). An alternative storyline approach might ask instead how much worse the event outcomes were because of the known thermodynamic aspects of climate change, such as ocean warming (Trenberth et al. 2015).

The distinction between thermodynamic and dynamic aspects of climate change is also useful when it comes to representing the uncertainty in projections of future change. A practical example of this is the KNMI Climate Change Scenarios (see http://www.climatescenarios.nl/), which provide four discrete sets of weather and sea-level variables, assuming a given global temperature increase and a regional amplification due to circulation responses (Van den Hurk et al. 2014). In essence, these provide storylines of regional climate given large-scale changes in physical climate.

On a continental scale, it is possible to make statements about future climate (such as extreme precipitation in the mid-latitudes will increase) with a high degree of confidence since the spatially aggregated signal is driven primarily by the thermodynamic response to radiative forcing and global temperature increase (Fischer et al. 2013). However, at a regional scale, mid-latitude precipitation extremes are strongly influenced by the dynamic response, and they could either increase or decrease depending on the changes in circulation (Fischer et al. 2013; Pfahl et al. 2017). Manzini et al. (2014) showed that much of the model spread in wintertime circulation changes over the northern extratropics is related to the model spread in remote drivers such as tropical warming, Arctic warming and stratospheric vortex change. Zappa and Shepherd (2017) used this framework to develop storylines of European circulation change, conditioned on a given level of global warming. That the influence of the remote drivers is causal is supported by evidence from seasonal prediction and from single-forcing model experiments. It was found that for central European wintertime storminess and wintertime Mediterranean drying, two important climate change impacts for Europe, the difference between the most extreme storylines was equivalent to that from several degrees of global mean temperature increase.

The uncertainty in long-term (e.g. centennial) global temperature increase (for a given greenhouse gas forcing) is mainly associated with that in climate sensitivity. The observed warming provides only a very weak constraint on long-term equilibrium warming or climate sensitivity, and its direct applicability is a matter of considerable current debate. A storyline approach attempts to articulate the much richer physical understanding of how climate processes change as climate warms (or cools under past climate changes) to provide a more mechanistic way of constraining the problem. Using physically developed storylines, Stevens et al. (2016) show that greater confidence in an overall net-positive cloud feedback or better reconstructions of tropical temperatures at the Last Glacial Maximum could be enough to raise the lower bound on climate sensitivity. Critical tests for the upper bound could include testing of recent arguments for a more modest aerosol forcing.

## Exploring the boundaries of plausibility

If climate projections could be reliably articulated as probability predictions (conditioned on scenarios of future greenhouse gas emissions), then the societal consequences could be presented as quantified risks or as changing risk profiles. The conventional approach to climate projection is focused on making such (conditional) probability predictions by applying complex post-processing methodologies (including bias correction and downscaling) to the output of GCMs. If the climate system was well understood and could be simulated accurately at all relevant scales, then this engineering approach to climate projections would be appropriate. However, such a proposition has been challenged (Smith 2002; McWilliams 2007; Van Oldenborgh et al. 2013). Furthermore, twenty-first century projections with GCMs have only partial exploration of model uncertainty (Knutti et al. 2013) and of initial condition uncertainty (Hawkins et al. 2016). Confidence in the processes simulated by climate models rests instead on the robustness of the model behaviour when compared with a variety of types of observations, within a causal framework (Lloyd 2015). As a consequence, there is a strong need to incorporate physical understanding of the processes of climate change directly into climate-related statements about the future (Maraun et al. 2017).

Storyline approaches can do this by placing the emphasis on physical plausibility when making statements about future climate. They do so by using models as tools to support and test theories regarding the interactions of climate processes, rather than as a source of quantitative climate predictions. Scientific understanding is used to push out the boundaries of plausible futures by proposing mechanisms through which outcomes not currently seen in GCMs, either because of missing physics or because of insufficient sampling, could arise (Hazeleger et al. 2015; Zscheischler et al. 2018). This provides a more thorough exploration of the range of uncertainty reflected by today’s level of scientific understanding, compared to the limited range represented by the extant ensembles of GCM projections.

The fact that climate models do not fully simulate the range of response uncertainty is particularly evident for local-scale extreme events. For example, it has been found that short-duration (hourly) summertime precipitation extremes are well simulated only in very-high-resolution (< 4 km) regional climate models (RCMs) that explicitly represent deep convection, and not in conventional RCMs that parameterise deep convection, and that intensity increases with warming are much larger in the high-resolution simulations (Kendon et al. 2017). This suggests that conventional national climate change scenarios may underestimate the potential changes to short-duration precipitation extremes. However, very-high-resolution regional simulations can be used together with observations and theoretical understanding to provide risk estimates within a storyline framework. Meredith et al. (2015) argued that a convective event leading to intense flooding could be attributed to Black Sea warming using an RCM with explicit convection constrained by large-scale reanalysis, but that the response simulated with parameterised convection was physically implausible. Prein et al. (2016) used a similar approach to make statements about future risk of extreme precipitation and flooding for the USA. Such targeted very-high-resolution simulations of relevant phenomena combine process understanding with the sampling of otherwise unexplored uncertainties and thereby greatly contribute to the establishment of robust projections of localised extreme events.

As an example of an application to climate impacts, UK Water Industry Research addressed the challenge of long-term change in intense storms by commissioning a study to build a plausible ‘story’ using the best scientific evidence of how these storms might evolve under global warming. The study used theoretical understanding of potential change in precipitation extremes from the Clausius-Clapeyron relation (7%/K) and examined climate analogues (using summer temperature and precipitation climatology as analogues) and outputs from models with explicit convection to produce plausible future scenarios, which were then translated into design guidance for storm drainage networks (Dale et al. 2017). In this case, the climate analogues, high-resolution model predictions and theory agreed on a common trajectory, which gave additional confidence in the results.

In the Netherlands, where sea level rise constitutes an existential risk, there has been a paradigm shift from predicting to exploring the future, which has increased the opportunity for robust decision-making (Haasnoot and Middelkoop 2012). The Delta Commission of the Netherlands addressed the challenge of high-end, long-range sea level change by deconstructing all the components of sea level rise and building plausible accounts of how each might evolve under extreme global mean warming (Katsman et al. 2011). In this study, each source of information, whether climate model output, palaeoclimatic data, observations or expert elicitation, is presented in a transparent and auditable format that is open to challenge and revision as scientific understanding grows. This field of expertise is developing rapidly, and frequently, new insights on major contributors to sea level rise are explored (e.g. DeConto and Pollard 2016), which trigger scientific and public debates on the severity of the risks for extreme sea level rise. Using a similar process-based decomposition method, Le Bars et al. (2017) presented projections of sea level rise exceeding those of Katsman et al. (2011), which triggered the Dutch Delta Programme to commission a new study on potential consequences of these projections for the safety of the Netherlands. Such a concept of ‘adaptive delta management’ as applied in the Netherlands is based on the principle that new insights or events may trigger the need to change the defence strategy, and relies heavily on its ability to monitor and digest new evidence and information offered by consecutive storylines of major climatic phenomena (Haasnoot et al. 2018).

## Conclusion

We have presented a typology of four reasons for taking a storyline approach in climate change research and communication. Storylines can raise risk awareness by framing risk in an event-oriented rather than a probabilistic manner—where events can be long-lasting, such as a trend—which corresponds more directly to how people perceive and respond to risk. Storylines can provide an effective mechanism for strengthening decision-making by allowing one to work backward from a particular vulnerability or decision point, combining climate change information with other relevant factors to address compound risk and develop appropriate stress tests. Storylines can provide a physical basis for partitioning uncertainty, thereby allowing the use of more credible regional models in a conditioned manner. Finally, storylines can explore the boundaries of plausibility, beyond what might be provided by a standard set of modelling tools, thereby guarding against false precision and surprise (Parker and Risbey 2015).

Although this paper has focused on the application of storylines to physical aspects of climate change, the same reasons for their use are relevant to other aspects of climate change where probabilistic modelling is widespread, e.g. economic models of climate damages. An important aspect of emerging storylines in this context is that they are path-dependent and endogenous. In other words, the impact of non-marginal climate shocks on human society is unlikely to be just temporary. Rather, such shocks alter the future dynamic course of human and environmental development by undermining or destroying physical, human and environmental capital—sometimes irreversibly. Dietz and Stern (2015) show that this has important implications for appropriately modelling storylines associated with the human impact of climate risks. Accounting for three essential elements of the climate problem—the endogeneity of growth, the convexity of damages, and climate risk—they argue, means optimal policy comprises much stronger controls, and earlier action to reduce emissions, than implied by conventional models based on implausibly simplified storylines.

The term ‘post-normal science’ was introduced by Funtowicz and Ravetz (1993) to describe a situation where either the decision stakes or the systems uncertainties are high, and hence the use of evidence is contested. They noted that many environmental issues fall into this category, including climate change. In such a situation, traditional methods of scientific problem-solving (reductionist, expert-based) are ineffective. The failure to recognise these limitations helps explain why there has been a persistent gap between the production and use of climate information (Kirchhoff et al. 2013).

According to Funtowicz and Ravetz (1993), post-normal science acknowledges unpredictability, incomplete control, and a plurality of legitimate perspectives. The goal is not to banish uncertainty, but to manage it. The model for scientific argument is not a formalised deduction, but an interactive dialogue. Storylines offer many possibilities in these respects, not least as ‘conversation starters’. More broadly, they provide a means of navigating the ‘cascade of uncertainty’ (e.g. Wilby and Dessai 2010) by representing uncertainty in physical aspects of climate change without losing sight of the robust aspects, whilst using concepts that relate to people’s experience and tap into their episodic way of thinking. Given their prevalence in consideration of the human dimension, storylines also offer a powerful way of linking physical with human aspects of climate change.
